# Incidence and risk factors for retinopathy of prematurity: a retrospective cohort study

**DOI:** 10.1186/s40942-018-0125-z

**Published:** 2018-05-31

**Authors:** André Moraes Freitas, Ricardo Mörschbächer, Mariana R. Thorell, Ernani Luis Rhoden

**Affiliations:** 1 Universidade Federal de Ciências Da Saúde de Porto Alegre (UFCSPA), Rua Sarmento Leite, 245, Porto Alegre, RS 90050-170 Brazil; 2 Royal Liverpool University Hospital, Prescot St, Liverpool, L7 8XP UK

**Keywords:** Premature birth, Extremely preterm infant, Neonate, Retinopathy of prematurity, Intensive care units, pediatric

## Abstract

**Background and objectives:**

Advances in neonatal care promoted increased survival rates of preterm infants, with a consequent increase in the number of children affected by retinopathy of prematurity (ROP). This study estimates the incidence of ROP and evaluates potential risk factors associated.

**Methods:**

A retrospective cohort study of preterm infants born in a tertiary neonatal intensive care unit was conducted from March 2005 to August 2015. Six hundred and thirty-nine newborns were included based on the following criteria: infants born with less than 32 weeks’ gestation or birth weight below 1500 g; or neonates born with 32–37 weeks’ gestation or birth weight above 1500 g and any of the following associated: multiple gestation, respiratory distress syndrome, sepsis, blood transfusions or intraventricular hemorrhage. Neonates were followed up until disease resolution or until treatment criteria was achieved.

**Results:**

A total of 602 newborns were evaluated after applying the exclusion criteria. Mean gestational age was 30.7 ± 2.5 weeks. The incidences of ROP at any stage and of type 1 prethreshold ROP were 33.9 and 5.0% respectively. Logistic regression analysis revealed that risk factors associated with ROP at any stage were extremely low birth weight (ELBW) (odds ratio [OR] = 3.10; 95% confidence interval [95% CI]:1.73–5.55), pulmonary diseases (OR = 2.49; 95% CI: 1.35–4.59), intraventricular hemorrhage (OR = 2.17; 95% CI: 1.10–4.30), and low gestational age (OR = 0.81; 95% CI: 0.73–0.91). The main risk factors associated with type 1 prethreshold ROP were pulmonary diseases (OR = 9.58; 95% CI: 1.27–72.04) and ELBW (OR = 3.66; 95% CI: 1.67–8.00).

**Conclusion:**

This study found a significant incidence of ROP (33.9%) in the studied population, and highlighted pulmonary diseases as a significant risk factor for type 1 prethreshold ROP.

## Background

Retinopathy of prematurity (ROP) is a condition characterized by the development of abnormal retinal vessels secondary to an incomplete vascularization of the retinal tissue due to hyperoxia causing downregulation of VEGF and death of endothelial cells. This mechanism suggests that VEGF plays a vital role for the endothelium. Following the closure of growing vessels, the retinal tissue in development becomes ischemic and hypoxic. This process upregulates VEGF leading to neovascularization [1–3]. The disease has been extensively studied worldwide due to increased survival rates among very low birth weight preterm newborns (NBs), i.e. with birth weight (BW) ≤ 1500 g, who are at the greatest risk for developing ROP. These increased numbers may be attributed to improved perinatal care. The high rates are leading to a significant raise in the occurrence of other comorbidities related to preterm birth [4] that have major social repercussion, such as blindness secondary to ROP. Worldwide, nearly 10% of all births are premature (before 37 weeks’ gestation) [5]. Blencowe et al. [6] estimated that every year 32,000 neonates became blind or developed severe visual impairment due to ROP worldwide, of which 10% were born in Latin American and the Caribbean. The risk factors associated with ROP were found to vary depending on the region [7]. These variations are possibly related to the heterogeneity of the population and discrepancy in neonatal care.

ROP is a multifactorial disease [8]. Many studies report several risk factors associated with this condition, some of which can cause severe ROP (BW, Gestational age (GA), supplemental oxygen, prolonged mechanical ventilation, Apgar score, pulmonary complications, anemia, intraventricular hemorrhage (IVH), necrotizing enterocolitis, sepsis) [2, 9–13]. The identification of risk factors interfering with the progression of ROP and the knowledge on its etiology may help ophthalmologists and neonatologists to perform careful screening, execute accurate diagnosis, and prevent development of the disease. The aim of this study was to estimate the incidence of ROP and to assess the association between ROP and potential risk factors for this condition, particularly type 1 prethreshold ROP, in newborns admitted to a neonatal intensive care unit (NICU) of a Brazilian tertiary hospital. Type 1 prethreshold ROP determines the need for treatment. It is defined as zone I any stage ROP with plus disease, or zone I stage 3 ROP without plus disease, or zone II stage 2 or 3 with plus disease [14].

## Patients and methods

This was a retrospective cohort study of preterm infants admitted in a tertiary neonatal intensive care unit from March 2005 to August 2015. The project was approved by the Research Ethics Committee of the Federal University of Health Sciences of Porto Alegre—UFCSPA (Certificate of Ethical Appreciation no. 45,477,615.4.0000.5335).

Inclusion criteria followed the guidelines proposed by the Brazilian Council of Ophthalmology and the Brazilian Society of Pediatric Ophthalmology, presented at the Workshop of ROP held by the Brazilian ROP Group in 2007 [14]. These guidelines defined the ROP screening criteria in Brazilian NICUs, and also recommended that NBs with prethreshold type 1 ROP should be treated.

Patients who died before complete resolution of ROP or did not attend the outpatient clinic for follow-up examinations were excluded from the study. Patients with incomplete data on BW or GA were also excluded, as well as those with congenital glaucoma or congenital cataract.

The study included preterm infants meeting one of the following criteria:

- BW ≤ 1500 g or GA ≤ 32 weeks; or
- BW > 1500 g or GA from 32 to 37 weeks and any of the following risk factors: respiratory distress syndrome (hyaline membrane disease), sepsis, blood transfusions, multiple pregnancy, intraventricular hemorrhage.

Fundoscopy was performed under mydriasis with one drop of 0.5% tropicamide eye drops combined with 1% phenylephrine eye drops instilled three times in each eye, with 15-minutes interval in between, before examination.

Retinal examination was performed at bedside using a binocular indirect ophthalmoscope (OSF 1.0 Eyetec, São Carlos, Brazil), a 20-diopter lens (Ocular Instruments Inc., Bellevue, WA, USA), a newborn eyelid speculum (Roca, São Paulo, SP, Brazil), and a pediatric scleral depressor (Roca, São Paulo, SP, Brazil). The examination was conducted by one of the authors (AMF), an experienced ophthalmologist with competent training in diagnosis and management of pediatric retinal diseases.

Each patient was classified according to the most advanced stage of ROP observed during follow-up assessment, considering the eye with more advanced disease, based on the International Classification of Retinopathy of Prematurity [15]. Patients were also classified as presenting type 1 prethreshold ROP or not, which determines the need for treatment, as established by the Early Treatment for Retinopathy of Prematurity (ETROP) randomized trial [16].

The first examination was performed between 4 and 6 weeks of chronological age. Subsequent examinations were scheduled according to Brazilian ROP Group guidelines, at intervals determined by the findings observed at each examination [14].

Data collection was discontinued when retinal vascularization was complete, reaching extreme temporal periphery, or when ROP showed complete regression after treatment. After that, patients were referred for routine follow-up assessment with a pediatric ophthalmologist at 6 months of age. Patients discharged from the NICU were scheduled for outpatient follow-up if reassessment was indicated as above.

Study variables were selected based on the Brazilian guidelines for screening and treatment of ROP [14]. We collected data on gender; BW; GA; Apgar scores at 1 and 5 min; number of days on any oxygen therapy or on continuous positive airway pressure (CPAP) or mechanical ventilation; number of blood transfusions; type of birth (singleton vs. multiple); occurrence of sepsis; use of surfactants; occurrence of IVH; use of erythropoietin; maternal use of antenatal corticosteroids; and presence of systemic comorbidities such as cardiac diseases (valvulopathy, interatrial or interventricular communication, patent ductus arteriosus), pulmonary diseases (pneumonia, bronchopulmonary dysplasia, or hyaline membrane disease), or digestive diseases (necrotizing enterocolitis or jejunal atresia).

Statistical calculations were performed using SPSS software version 22.0, and R software version 3.3.0. Normally distributed quantitative data were expressed as mean and standard deviation (SD). Asymmetrically distributed variables were represented as median, minimum, and maximum values. Categorical variables were reported as counts and percentages. Initially, comparisons were made between patients with and without ROP. Subsequently, patients with type 1 prethreshold ROP, i.e., requiring treatment, were compared with the remaining patients. Quantitative data were analyzed using the Student’s t test for normally distributed variables or the Mann-Whitney U test for asymmetrically distributed variables. Categorical data were assessed using the Chi square test. A forward stepwise logistic regression model was used to estimate the association between risk factors and development of ROP and type 1 prethreshold ROP in order to adjust for potential confounding factors.

In an attempt to minimize bias, a statistical rule of thumb suggests that at least 10 cases of the rarest outcome event are required per each variable to be included in a logistic regression model [17]. Since 30 neonates developed type 1 prethreshold ROP in our study, we decided to include three variables in the logistic regression model to evaluate the potential risk factors associated with this outcome. These variables were selected among the four variables found to be associated with the development of ROP at any stage, namely BW < 1000 g, GA, presence of IVH, and pulmonary comorbidities. Of these, we decided to exclude GA, because a strong correlation was observed between this variable and BW and because GA is a less accurate measure than BW. GA is not always an accurate measurement because it is estimated based on the first day of the last menstrual period, which may be influenced by recall bias, and it is also estimated by obstetric ultrasound [18]. Therefore, BW  < 1000 g, presence of IVH, and pulmonary comorbidities were included in the final logistic regression model. Finally, the logistic probability of occurrence of type 1 prethreshold ROP was calculated including BW and pulmonary comorbidities. Statistical significance was set at *P* < 0.05.

## Results

During the study period, 639 patients met the inclusion criteria. Of these, 37 newborns were excluded from the study analysis: 25 failed to attend outpatient ophthalmological follow-up visits, 9 died before ROP was resolved, and 3 had incomplete records. A total of 602 patients remained in the study, with mean GA of 30.7 ± 2.5 weeks and mean BW of 1274 ± 385 g. Of these, 302 (50.2%) were male. A total of 204 patients presented with ROP (incidence of 33.9%). Of these, 160 (26.6%) developed stage 1 ROP; 26 (4.3%), stage 2 ROP; and 18 (3%), stage 3 ROP. None of the patients developed stages 4 and 5 ROP. Thirty patients (5.0%) developed type 1 prethreshold ROP and required treatment.

In the overall sample, there were 520 neonates with GA below 32 weeks or with BW below 1500 g, of which 196 (37.6%) developed ROP. All 30 patients who developed type 1 prethreshold ROP belonged to this subgroup. Eight patients with GA above 32 weeks and BW above 1500 g developed ROP, all of those classified as stage 1.

Table 1 shows results of bivariate analysis comparing patients with and without ROP. Statistically significant differences were observed between the groups for all variables, except for gender, type of birth, and presence of digestive diseases. Multivariate forward stepwise logistic regression analysis revealed that the risk factors associated with the development of ROP at any stage were BW < 1000 g, lower GA, occurrence of IVH, and presence of lung diseases (pneumonia, bronchopulmonary dysplasia, or hyaline membrane disease).

**Table 1:** Risk factors for the development of retinopathy of prematurity (ROP) at any stage

Risk factors	ROP	Without ROP	*P*
	*n* = 204 (33.9%)	*n* = 398 (66.1%)	
Male, *n* (%)	101 (49.5%)	201 (50.5%)	.689^a^
Type of birth, *n* (%)			.832^a^
Singleton	169 (82.8%)	326 (81.9%)	
Twin or multiple	35 (17.2%)	72 (18.1%)	
Sepsis, *n* (%)	167 (83.5%)****	285 (71.6%)	.001^a^
Use of surfactants, *n* (%)	179 (88.6%)**	258 (65%)*	< .001^a^
Intraventricular hemorrhage, *n* (%)	54 (26.7%)**	50 (12.6%)	< .001^a^
Erythropoietin, *n* (%)	16 (7.9%)**	12 (3%)	.006^a^
Maternal use of antenatal corticosteroids, *n* (%)	58 (28.7%)**	44 (11.1%)	< .001^a^
Cardiac diseases, *n* (%)	69 (33.8%)	51 (12.8%)	< .001^a^
Pulmonary diseases, *n* (%)	171 (83.8%)	250 (62.8%)	< .001^a^
Digestive diseases, *n* (%)	11 (5.4%)	16 (4%)	.577^a^
Birth weight < 1000 g, *n* (%)	98 (48.0%)	50 (12.6%)	< .001^a^
Gestational age (weeks), mean ± SD	29.4 (2.5)	31.4 (2.2)	< .001^b^
Apgar score at 1 min, mean ± SD	6 (2.3)	6.7 (2.2)	.001^b^
Apgar score at 5 min, mean ± SD	7.7 (1.4)	8.2 (1.5)	< .001^b^
Days on oxygen therapy, median (minimum to maximum)	27 (0–150)	6 (0–150)	< .001^c^
Days on CPAP, median (minimum to maximum)	4 (0–42)	2 (0–30)	< .001^c^
Days on mechanical ventilation, median (minimum–maximum)	7 (0–103)	1 (0–150)	<.001
Number of blood transfusions, median (minimum to maximum)	1 (0–15)	0 (0–12)	< .001

*CPAP* continuous positive airway pressure

*1 newborn with missing data; **2 newborns with missing data; ***3 newborns with missing data; ****4 newborns with missing data

^a^chi-square test

^b^Student t test

^c^Mann-Whitney U test

Table 2 shows results of bivariate analysis comparing patients with and without type 1 prethreshold ROP. Statistically significant differences were observed between the groups for all variables, except for gender, type of birth, presence of IVH, use of erythropoietin, maternal use of antenatal corticosteroids, and digestive diseases.

**Table 2:** Risk factors for the development of retinopathy of prematurity (ROP) with indication for treatment

Risk factors	With type 1 prethreshold ROP	Without type 1 prethreshold ROP	*P*
	*n* = 30 (5%)	*n* = 572 (95%)	
Male, *n* (%)	12 (40%)	290 (50.7%)	.267^a^
Type of birth, *n* (%)			.805^a^
Singleton	24 (80%)	470 (82.5%)**	
Twin or multiple	6 (20%)	100 (17.5%)	
Sepsis, *n* (%)	28 (96.6%)*	424 (74.5%)***	.004^a^
Use of surfactants, *n* (%)	29 (100%)*	408 (71.6%)**	< .001^a^
Intraventricular hemorrhage, *n* (%)	9 (30%)	95 (16.7%)**	.79^a^
Erythropoietin, *n* (%)	2 (6.7%)	26 (4.6%)**	.644^a^
Antenatal corticosteroids, *N* (%)	8 (27.5%)*	94 (16.5%)*	.211^a^
Cardiac diseases, *n* (%)	12 (40%)	105 (18.4%)	.007^a^
Pulmonary diseases, *n* (%)	29 (96.7%)	392 (68.5%)	< .001^a^
Digestive diseases, *n* (%)	3 (10%)	24 (4.2%)	.146^a^
Birth weight < 1000 g, *n* (%)	18 (60.0%)	130 (22.7%)	< .001^a^
Gestational age (weeks), mean ± SD	28.5 (2.6)	31.8 (2.4)	< .001^b^
Apgar score at 1 min, mean ± SD	5.6 (2.43)	6.5 (2.2)	.006^b^
Apgar score at 5 min, mean ± SD	7.2 (1.6)	8.1 (1.4)	< .001^b^
Days on oxygen therapy, median (minimum to maximum)	41 (2–150)	8 (0–150)	< .001^c^
Days on CPAP, median (minimum to maximum)	4 (0–42)	2 (0–34)	< .001^c^
Days on mechanical ventilation, median (minimum to maximum)	16 (0–60)	2 (0–150)	< .001^c^
Number of blood transfusions, median (minimum to maximum)	2 (0–15)	1 (0–14)	< .001^c^

*CPAP* continuous positive airway pressure

*1 newborn with missing data; **2 newborns with missing data; ***3 newborns with missing data

^a^chi-square test

^b^Student t test

^c^Mann-Whitney U test

**Fig. 1:**
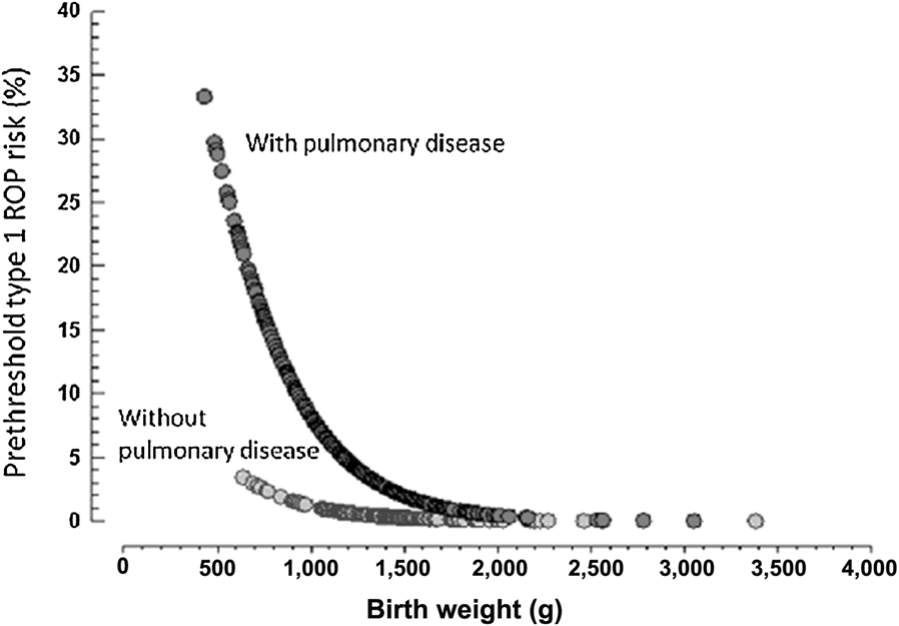
Risk for type 1 prethreshold retinopathy of prematurity by a logistic regression model. In patients without pulmonary diseases, progressively lower BW lead to a increase in the risk of developing type 1 prethreshold ROP, however, in lower percentage when compared to the group with pulmonary diseases

Among the most relevant variables for the comparison between groups with ROP and without ROP (BW < 1000 g, occurrence of IVH, and pulmonary diseases), pulmonary diseases and BW < 1000 g remained significant risk factors for type 1 prethreshold ROP after logistic regression analysis (Table 3). Logistic regression analysis revealed that the lower the BW, the higher the risk for the development of type 1 prethreshold ROP, especially in the presence of pulmonary diseases (Fig. 1).

**Table 3:** Risk factors for the development of retinopathy of prematurity (ROP): results of logistic regression analysis

Risk factor	ROP at any stage *n* = 204			Type 1 prethreshold ROP *n* = 30		
	OR	95% CI	*P*	OR	95% CI	*P*
Birth weight < 1000 g	3.10	1.73–5.55	< .001	3.66	1.67–8.00	.001
Pulmonary diseases	2.49	1.35–4.59	.004	9.58	1.27–72.04	.028
Intraventricular hemorrhage	2.17	1.10–4.30	.026	1.61	0.69–3.79	.27
Gestational age	0.81	0.73–0.91	.003			

*OR* odds ratio, *95% CI* 95% confidence interval

## Discussion

Table 4 summarizes a comparison between ROP studies. ROP incidence varies considerably, reflecting the differences in screening criteria, neonatal care and population heterogeneity. Some risk factors are well stablished (GA, BW), but there is no clear consensus about the others.

**Table 4:** Studies on the incidence and risk factors for ROP

Author	Country	Year of publication	Study design	Patients (n)	Inclusion criteria	Incidence of any stage ROP (%)	Main risk factors
Schaffer et al. (CRYO-ROP Study) [31]	United States	1993	MRCT	4099	BW < 1251 g	66.0	GA, BW, multiple births, Out of nursery birth, white race
Good et al. (ET-ROP Study) [16]	United States	2004	MRCT	6998	BW < 1251 g	68.0	NR
Fortes Filho et al. [4]	Brazil	2009	PCS	450	BW < 1500 g or GA < 32 w	24.2	GA, BW, PMV, sepsis, IVH, BT
Zin et al. [23]	Brazil	2010	PCS	3437	BW < 2000 g or GA < 37 w	19.9	NR
Lomuto et al. [32]	Argentina	2010	RCS	956	BW < 1500 g or GA < 32 w	26.2	NR
Gonçalves et al. [11]	Brazil	2014	PCS	110	BW < 1500 g or GA < 32 w	44.5	GA, BW, BT, sepsis
Mitsiakos et al. [33]	Canada	2016	RCS	1562	GA < 32 w	15.6	BW, sepsis, NEC, PDA, PMV
Yau et al. [34]	China	2016	RCS	513	BW < 1500 g or GA < 32 w	18.5	GA, BW, IVH
Owen et al. [35]	United States	2017	RCS	457	BW < 1250 g or GA < 30 w	47.5	GA, BW, maternal Mg prophylaxis, need of any surgery
Ahuja et al. [36]	India	2018	PCS	325	BW < 1900, GA < 36 w	32.6	BW
Bas et al. [37]	Turkey	2018	PCS	6115	BW < 1500 g, or GA < 32 w, or unstable clinical course	27.0	GA, BW, days on oxygen, sepsis, BT, relative weight gain

*ROP* retinopathy of prematurity, *MRCT* multicenter randomized clinical trial, *PCS* prospective cohort study, *RCS* retrospective cohort study, *g* grams, *w* weeks, *NR* not reported, *GA* gestational age, *BW* birth weight, *PMV* prolongued mechanical ventilation, *IVH* intraventricular hemorrhage, *BT* blood transfusions, *NEC* necrotizing enterocolitis; *PDA* persistent ductus arteriosus, *Mg* magnesium

Many ROP studies screened only infants born with less than 32 weeks’ gestation or with less than 1500 g of BW [4, 11]. In our study, however, we went further and also included infants with more than 1500 g or more than 32 weeks’ gestation with determined risk factors associated. Our results showed that the incidence of ROP at any stage was 33.9% while the incidence of type 1 prethreshold ROP was 5.0%. Similarly, in a subgroup analysis of 520 newborns based on the above criteria (infants born at less than 32 week’’ gestation or with less than 1500 g), 37.6% developed ROP at any stage and 5.7% developed type 1 prethreshold disease. This subgroup included all 30 subjects requiring treatment. However, eight patients in the study who developed stage 1 ROP did not belong to this subgroup. The incidences of ROP at any stage and of type 1 prethreshold ROP in this subgroup were at intermediate levels of the range reported in Brazilian studies that used the same selection criteria. Fortes Filho et al. [4] found an incidence of ROP at any stage and of type 1 prethreshold ROP of 29.6 and 7% respectively. Conversely, Gonçalves et al. [11] found an incidence of ROP at any stage and of type 1 prethreshold ROP of 44.5 and 1.8% respectively. The use of various inclusion criteria for patients in the screening programs performed in different Latin American countries limits any further comparative analysis of the published data.

The Vermont Oxford Network database, which collects data from more than 1000 NICUs worldwide, estimated in 2010 an incidence of 33.2% of ROP in neonates with BW < 1500 g [19]. These variations in the incidence of ROP may reflect differences in study populations, mortality rates, and characteristics of neonatal care in each institution, corroborating the need to further investigate the risk factors for the development of ROP. Numerous published case series have shown that infants with ROP in low- and middle-income nations have higher average BW and GA than infants with ROP in the United States [20]. Most studies report ROP incidences of about 60% for babies with less than 1500 g in nurseries of high-income countries [21]. In middle-income countries, this scenario is significantly variable depending on the birth conditions and survival rates of premature infants, and due to the fact that ROP occurs in much older and bigger babies than in high-income countries because of varying standards of neonatal care [21].

The identification of at-risk preterm infants is important for establishing screening criteria, in order to avoid unnecessary ophthalmological examinations and to ensure the assessment of every premature newborn who develops severe ROP. Many studies have been seeking ways of optimizing screening for ROP by considering postnatal weight gain in mathematical models to help identify at-risk preterm infants [12, 13, 22]. Some studies have demonstrated that it is possible to reduce the number of unnecessary examinations by using methods with good sensitivity and specificity [22, 23]. However, these methods have been suggested to assist in selecting and monitoring preterm infants but not to replace traditional selection criteria for patient screening; hence, they may be validated in other studies. Looking into this, in 2010 Zin et al. [23] published a study evaluating methods for selecting NBs to be screened for ROP in seven NICUs in Rio de Janeiro, southeastern Brazil. Results suggest that, in NICUs with lower survival rates, selection criteria should be expanded to NBs with 35 weeks’ gestation in order to identify patients with ROP requiring treatment.

In our 10-year study, all NBs requiring treatment met the criteria for GA below 32 weeks and BW below 1500 g. Preterm infants with other risk factors and born with less than 37 weeks’ gestation were included to ensure a safety margin for patient selection, since none of the preterm infants with BW and GA above the previously mentioned values developed ROP requiring treatment. If screening was based strictly on BW and GA, 82 patients (13.6%) would not undergo examination. However, it is important to note that GA is not always an accurate measurement, because it is estimated based on the last menstruation date, which may be influenced by recall bias, and depends on the availability of obstetric ultrasound. It is also worth mentioning that eight preterm infants born at more than 32 weeks’ gestation and with BW > 1500 g developed stage 1 ROP.

Many studies found that the lower the BW and the GA, the greater the risk of developing ROP [2, 4, 9, 24–26]. These findings were corroborated by our logistic regression analysis. Besides these variables, the occurrence of IVH and the presence of pulmonary diseases also remained significant in the logistic regression model.

The presence of IVH has been associated with severity of ROP [26, 27]. The development of that condition is related to hypoperfusion and cerebral hypoxia in preterm infants and to the rupture of immature subependymal vessels [27]. Therefore, IVH and ROP are both characterized by tissue ischemia and vascular immaturity. Our results corroborate the recommendation of Brazilian guidelines for screening and treatment of ROP, which point out IVH as an important risk factor to be considered in the selection of patients to be assessed [14].

The presence of pulmonary diseases was an important risk factor for the development of both ROP at any stage and type 1 prethreshold ROP. This observation suggests that pulmonary diseases may be used as markers for the need of larger amount of supplemental oxygen, which is known to be essential in the pathogenesis of ROP [28]. In addition, pulmonary diseases are also possibly associated with fluctuations in oxygen concentration and episodes of intermittent hypoxia that are also related to a greater risk of developing ROP [29].

The logistic regression model revealed that pulmonary diseases and BW < 1000 g are the main risk factors for the development of type 1 prethreshold ROP. BW as a continuous variable and the presence of pulmonary diseases were used to calculate the logistic probability of development of type 1 prethreshold ROP. Figure 1 shows that the lower the BW, the greater the probability of type 1 prethreshold ROP. This figure also illustrates that the association between low BW and pulmonary diseases is a determining factor in the increase of this probability. This figure demonstrates that, in patients without pulmonary diseases, progressively lower BW lead to an increase in the risk of developing type 1 prethreshold ROP, however, in lower percentage when compared to the group with pulmonary diseases.

The aim of this study was not to assess the outcome of patients with type 1 prethreshold disease, but it is worth mentioning that they were all treated with diode laser photocoagulation and showed complete regression of ROP.

The limitations of the present study include its retrospective nature limiting the control over the quality of measurements. It should also be taken into account that we have included neonates born over a 10-year period, when changes in neonatal care may have been implemented. Nevertheless, the advantages of the study, which ensured its clinical importance, include a robust sample size, the assessment of the disease under study by a single trained examiner, and the availability of intensive care resources at a tertiary hospital serving a large population of a metropolitan area. Furthermore, a detailed statistical analysis was determinant to reduce study bias.

Although a variety of instruments have been available to image premature neonates, we did not have access to imaging devices during the course of the study. Objective documentation of the disease requires a trained imager with skills to obtain images of satisfactory quality. Expert grading is accurate and used to compare with imaging graders in several studies [30].

## Conclusion

Although the incidence of ROP in our study was at intermediate levels compared to Brazilian studies with analogous design, our mean incidence was very similar to worldwide results, as the Vermont Oxford Network database. Our study contributed to the analysis of risk factors associated with ROP, supporting the importance of low BW, low GA, and IVH. Our results revealed that impaired lung function as shown by the occurrence of pneumonia, hyaline membrane disease, or bronchopulmonary dysplasia, is an important risk factor for the development of ROP and a determinant element of increased risk of developing type 1 prethreshold ROP. This stage of ROP maintains the greatest interest, because it establishes the need of therapeutic intervention. To the best of our knowledge, this is the first study reporting the association between pulmonary diseases and type 1 prethreshold ROP. Further prospective studies assessing these variables are required in order to evaluate the magnitude of the cause-effect relationship between pulmonary diseases and severe ROP.

## Abbreviations

ROP: retinopathy of prematurity
BW: birth weight
CPAP: continuous positive airway pressure
ELBW: extremely low birth weight
ETROP: early treatment for retinopathy of prematurity
GA: gestational age
IVH: intraventricular hemorrhage
NB: newborn
NICU: neonatal intensive care unit
